# Major and trace mineral composition of milk from lactating women following vegan, vegetarian and omnivore diets

**DOI:** 10.1017/S0007114522004007

**Published:** 2023-09-28

**Authors:** Maryanne T. Perrin, Roman Pawlak, Nicholas Judd, Jessica Cooper, George L. Donati

**Affiliations:** 1 Department of Nutrition, University of North Carolina Greensboro, 319 College Ave, 318 Stone Building, Greensboro, NC 27412, USA; 2 Department of Nutrition Science, East Carolina University, Health Science Building, Greenville, NC 27858, USA; 3 Department of Chemistry, Wake Forest University, Salem Hall, Winston-Salem, North Carolina 27109, USA

**Keywords:** Human milk, Breast milk, Plant-based diet, Selenium

## Abstract

Approximately one-in-ten reproductive age adults in the USA follow a plant-based diet, yet there is limited information on the influence of vegan and vegetarian diets on the mineral composition of breast milk. This study explored the major and trace mineral composition in breast milk and associations with maternal diet patterns. We used a cross-sectional design to collect a single sample of breast milk from individuals following vegan (*n* 23), vegetarian (*n* 19) and omnivore (*n* 21) diet patterns. Plant-based diet (*n* 42) was defined as following either vegan or vegetarian diets. Sixteen minerals were assessed using inductively coupled plasma mass spectrometry and inductively coupled plasma optical emission spectrometry. Data were evaluated using traditional statistical techniques and five different machine learning approaches. The distribution of Se (median; quartile 1 and 3) was significantly different between groups (vegetarians 21, 18–26 µg/l; vegans 19, 18–25 µg/l and omnivores 17, 14–20 µg/l; *P* = 0·007) using a Kruskal–Wallis test. Machine learning techniques also identified Se as a potential biomarker for differentiating breast milk by maternal diet pattern. Individuals following a plant-based diet generally had a lower BMI, higher breast milk Se and lower breast milk I and Fe concentrations compared with those following omnivore diets. This suggests that maternal dietary pattern (plant-based *v*. omnivore) may be helpful clinical information to consider when caring for the breast-feeding dyad, with the strongest evidence related to differences in Se concentration.

A recent Gallup poll reported that 8 % of Americans followed a vegetarian or vegan diet, with higher rates in reproductive age groups (10 % among ages 18–29 and 12 % in ages 30–49)^(1)^. Prior research suggests that adults following vegan or vegetarian diets may consume low amounts of some minerals including Ca, I, Se and Zn^(2–6)^. Among pregnant women, individuals following a vegetarian diet have significantly lower Zn intake and status compared with those following omnivore diets^(7)^. In an acknowledgment of these trends, the National Institute of Health has highlighted the need for more research to understand the impact of vegan and vegetarian diet patterns during lactation on the composition of human milk^(8)^.

Research regarding minerals in breast milk from vegan and vegetarian mothers is scarce, with only two studies identified in a recent review by Karcz *et al.* ^(9)^ A semi-longitudinal study by Finley *et al.* looked at well-nourished omnivore and vegetarian mothers (*n* 52) from 1 to 20 months postpartum and reported that breast milk Fe, K, Mg, Ca, Zn and Na were not influenced by maternal intake^(10)^. The authors noted that supplements contributed to the mineral intake of vegetarian mothers which could have explained some of the null findings. Debski *et al.* conducted a study of lacto-ovo-vegetarians (*n* 26) and omnivore (*n* 12) lactating women in California. While no difference in Se intake between the vegetarian and omnivore mothers was observed, there were significant differences in breast milk Se (22·2 (sd 0·8) ng/ml and 16·8 (sd 1·3), respectively; *P* < 0·01)^(11)^. It is important to note that the authors did not report the lactation stage which may have contributed to the findings, as Se has been observed to decrease in human milk over the first months postpartum^(12)^. With evidence of differing intake of some minerals when following a vegetarian or vegan diet that may translate into differences in breast milk composition, the purpose of this study was to explore relationships between vegan, vegetarian and omnivore diets during lactation and the concentration of 16 minerals in human milk, controlling for other factors including lactation stage (infant age) and the use of multi- or prenatal vitamins.

## Methods

### Study overview

We conducted a cross-sectional study that enrolled lactating women following vegan, vegetarian or omnivore dietary patterns. Se was selected to perform a power calculation based on reports of low intake among adults following a vegan *v*. an omnivore diet^(3)^. Using descriptive statistics for Se in breast milk of vegetarians *v*. non-vegetarians reported by Debski *et al.*, unbalanced groups of nineteen vegetarian and twenty-one omnivore diet and an *α* of 0·05, this study had a power of 0·97 for detecting differences in breast milk Se^(11)^. Detailed methods for study recruitment and sample collection have previously been reported^(13,14)^. Briefly, participants were lactating individuals who resided in the USA and consented to provide a single breast milk sample and complete a dietary screener to determine their diet pattern. The dietary screener asked about the consumption frequency of five different food groups: meat (including beef, lamb, pork, poultry); dairy products (milk, cheese and yogurts); eggs; fish and *n*-3 containing margarine, as well as the use of multi-micronutrient or prenatal supplements. Frequency responses were never; rarely (less than 1 time per month); sometimes (1–4 times/month) and often (more than 4 times/month). Participants were classified as vegan if they never consumed meat and fish and consumed non-meat animal products (e.g. dairy products, eggs) less than monthly (never or rarely). Vegetarians were characterised as not eating meat but regularly consuming other animal-based products. When vegan and vegetarian were combined into a single group for analysis, this was classified as a plant-based diet. Exclusion criteria included being < 2 weeks postpartum, being pregnant and known health conditions that could influence metabolism or B-12 status (e.g. gene mutation of methylene tetrahydrofolate reductase, hypo- or hyperthyroidism, celiac disease, liver disease). Milk samples were collected in the morning by complete expression from a single breast, stored in a breast milk storage bag and frozen until transport to the research lab.

### Sample analysis

Mineral concentrations in milk were determined by inductively coupled plasma mass spectrometry (ICP-MS) and inductively coupled plasma optical emission spectrometry (ICP-OES), using an Agilent 8800 ICP-MS/MS and an Agilent 5110 ICP-OES (Agilent Technologies). Helium or hydrogen gas, flowing at 3·5 or 4·0 ml/min, respectively, was used in the ICP-MS collision–reaction cell to minimise spectral interferences. Additional details on the operating conditions used in ICP-MS and ICP-OES determinations are summarised in online Supplementary Table S1. Milk samples were prepared according to a procedure adapted from Dubascoux *et al.* ^(15)^, which consisted of adding 0·30 ml (0 ml for ICP-OES) of distilled–deionised water (18 MΩ·cm, Purelab Option-Q, Elga) and 4·50 ml (4·98 ml for ICP-OES) of an aqueous solution (AlkS) containing 1 % v/v ammonia (NH_4_OH, Suprapur™, Millipore Sigma), 1 % v/v 2-propanol (Semiconductor grade, Alfa Aesar), 0·1 % m/v ethylene diamine tetra-acetic acid (EDTA, Electrophoresis grade, Alfa Aesar) and 5 × 10^−4^ % v/v Triton X-100 (Electrophoresis grade, Sigma Chemical Co.) to 0·20 ml (20·0 µl for ICP-OES) of milk. Blank solutions contained 0·50 ml (20·0 µl for ICP-OES) of distilled–deionised water and 4·50 ml (4·98 ml for ICP-OES) of AlkS. Calibration solutions were prepared with 9·0 ml (8·0 ml for ICP-OES) of AlkS and adequate volumes of standard reference solutions of As, Ca, Cd, Cr, Cu, Fe, I, K, Mg, Mn, Mo, Na, P, Pb, se and Zn (1000 or 10 mg/l, High Purity Standards) and distilled–deionised water for a final volume of 10·0 ml. Ca, K, Mg, Na and P were determined by ICP-OES, while the other elements were determined by ICP-MS. The analytical method was validated by addition and recovery experiments employing two different human milk samples. Spike concentrations were chosen based on a preliminary semi-quantitative analysis of the samples. For As, Cd, I, Mn, Mo, Pb and se, a concentration spike of 20 µg/l was adopted, while 40 µg/l was used for Cr, Cu, Fe and Zn. For Ca, K, Mg, Na and P, a spike concentration of 4 mg/l was chosen.

### Statistical analysis

A Shapiro–Wilk test was used to evaluate data normalcy, and a Levene test was used to evaluate homogeneity of variance. Only maternal age, K and Mg had normal distributions; all variables had homogeneous variance (*P* > 0·05) except lactation stage and diet duration. We used traditional statistical methods, as well as five different machine learning techniques to explore relationships between breast milk mineral composition and maternal diet pattern. The use of machine learning techniques overcomes some limitations associated with small sample sizes and facilitates the identification of hidden patterns in the data. Considering the relatively small data set, we have used multiple statistical methods to confirm variables associated with different maternal diet patterns^(16)^. Descriptive statistics were used to describe mineral composition in the full data set; correlation coefficients were computed to look for bivariate relationships between minerals and categorical variables were evaluated using a Fisher’s exact test. Statistical and machine learning techniques used to probe for differences between diet groups included: (i) Kruskal–Wallis analysis with a Dunn’s test for multiple comparisons using Holm’s procedure to adjust *P* values for non-parametric data and ANOVA with Tukey’s test for normally distributed data; (ii) random forest feature importance (RFI), which is a supervised, model-based embedded feature selection method based on random forests and the Gini index to identify the most important variables to split the data into different classification groups (in this case maternal diet)^(17,18)^; (iii) Boruta, which is a supervised feature selection wrapper based on random forest classification and backward feature elimination that ranks variables responsible for classifying the study samples into the different diet groups^(19)^; (iv) ReliefF, which is a supervised feature selection filter based on the k nearest neighbours technique to identify variables responsible for distingushing maternal diet patterns^(20,21)^; (v) support vector machine recursive feature elimination (SVM-RFE), which ranks the different variables according to their importance for maternal diet classification and assigns a *P*-value (at the 95 % confidence level) to each of them^(22,23)^ and (vi) penalised logistic regression with Lasso penalty, which shrinks the less contributive variables to zero and identifies the most relevant features within each diet group^(24,25)^.

Elements with more than 50 % of the samples presenting a concentration lower than the analytical method’s limit of quantification were removed from the data set (i.e. As, Cd, Cr and Mo). For the remaining elements, based on Succop *et al.*, entries with concentration values below the limit of detection were kept as they were^(26)^. For entries with a value of zero, a random number between zero and the limit of detection was employed. The R packages used for machine learning analyses were caret and randomForest for RFI; Boruta; caret and CORElearn for ReliefF; sigFeature for SVM-RFE and glmnet for penalised logistic regression with Lasso penalty.

Statistical analysis was conducted using SAS 9.4 (SAS Software) and the R programming language^(27)^ (R Foundation for Statistical Computing). This study was conducted according to the guidelines laid down in the Declaration of Helsinki, and all procedures involving human subjects/patients were approved by the the Institutional Review Boards at the University of North Carolina Greensboro and East Carolina University (16-0310 and 16-001726, respectively). Consent forms were provided to all participants via email, and written consent was waived by the Institutional Review Boards due to minimal risk of participation.

## Results

Seventy-four subjects completed the original study, and milk samples from sixty-three participants were available for mineral analysis based on the following maternal diet classification: vegan (*n* 23), vegetarian (*n* 19) and omnivore (*n* 21). Information about study participants is summarised in Table 1. There were no differences by diet group in maternal age, gravida or use of a multi-micronutrient or prenatal supplements. There were differences (median, quartile 1 to quartile 3) in BMI (vegans 22·0, 20·6–23·6; vegetarian 22·4, 21·1–25·0; omnivores 26·3, 22·2–28·0; *P* = 0·022), infant age at the time of sample collection (vegans 25, 14–46 weeks; vegetarians 50, 20–93 weeks; omnivores 25, 18–34 weeks; *P* = 0·031) and diet duration (vegans 5, 2–10 years; vegetarian 6, 3–9 years; omnivore 30, 25–33 years; *P* < 0·001). Multi-micronutrient or prenatal supplement use was reported by most study participants (41/63; 65 %) but did not differ statistically by maternal diet pattern (17/23, 74 % for vegans; 10/19, 52 % for vegetarians; 14/21, 67 % for omnivores; *P* = 0·38).

**Table 1.** Characterisation of study participants (Median and quartiles; mean values and standard deviations)

			By maternal diet pattern						
	Overall (*n* 63)		Vegan (*n* 23)		Vegetarian (*n* 19)		Omnivore (*n* 21)		
	Median	Quartile 1, quartile 3	Median	Quartile 1, quartile 3	Median	Quartile 1, quartile 3	Median	Quartile 1, quartile 3	*P*
Age (years)*									
Mean	32		33		33		32		0·81
sd	5		5		5		4		
BMI	22·5	21·1, 26·3	22·0^a^	20·6, 23·6	22·4^a^	21·1, 25·0	26·3^b^	22·2, 28·0	0·022
Infant age (weeks)	28	17, 48	25^a^	14, 46	50^a^	20, 93	25^a^	18, 34	0·031
Gravida	2	1, 3	2	1, 3	2	1, 3	2	2, 3	0·50
Diet Duration (years)	7	3, 25	5^a^	2, 10	6^a^	3, 9	30^b^	25, 33	<0·001
	Mean	sd	Mean	sd	Mean	sd	Mean	sd	
Race/Ethnicity† (%)									0·65
Asian	1	2	1	4	0	0	0	0	
White	53	84	19	83	17	90	17	81	
Hispanic	2	3	1	4	1	5	0	0	
Mixed	7	11	2	9	1	5	4	19	
Multi-micronutrient supplement use† (%)	41		17		10		14		0·38
%	65 %		73·9 %		52·6 %		66·7 %		

Data represent median (quartile 1, quartile 3) analysed using a Kruskal–Wallis test, unless otherwise noted. Values with a different superscript were significantly different using Dunn’s test for multiple comparisons.

* Data represent mean and standard deviation analysed using ANOVA.

† Data represent number (prevalence) of participants reporting use of a multi- or prenatal vitamin; differences evaluated using Fisher’s exact test.

### Overview of milk mineral composition

Both ICP-MS and ICP-OES methods applied to determine mineral concentrations in breast milk presented adequate accuracy. Analyte percent recoveries for all elements evaluated were in the 93–108 % range, with relatively low standard deviations (online Supplementary Table S2). Adequate limit of detection and limit of quantification values were also obtained for determinations in the original breast milk sample (i.e. before any dilution) for both analytical methods, with values in the 0·1–80 µg/l and 0·07–30 mg/l for ICP-MS and ICP-OES, respectively (online Supplementary Table S3). More than 50 % of the samples had mineral concentrations below the limit of quantification for arsenic, cadmium, chromium and molybdenum. Concentrations of these minerals and the number of samples above the limit of quantification were as follows: arsenic (1–11 µg/l; *n* 9); cadmium (1–4 µg/l; *n* 3); chromium (3–23 µg/l; *n* 12) and molybdenum (1–20 µg/l; *n* 19). The final statistical and machine learning analysis included twelve minerals: Ca, Cu, Fe, I, K, Mg, Mn, Na, P, Pb, Se and Zn.

Descriptive statistics for minerals in breast milk for the entire study population are presented in Table 2. Infant age was weakly associated with Se (*r* = 0·472; *P* < 0·001) and Zn (*r* = –0·486; *P* < 0·001). The minerals that had the least variability (min, max) were Ca (151–375 mg/l), K (270–548 mg/l) and P (75–190 mg/l). There was a greater than 10-fold difference in five minerals: Cu (1–3132 µg/l), Pb (0–88 µg/l), I (18–1719 µg/l), Mn (1–13 µg/l) and Zn (80–2312 µg/l). Significant correlations between minerals were mostly moderate (*r* = 0·4–0·69)^(28)^ and included Pb and Mn (*r* = 0·732; *P* < 0·001); Se and Na (*r* = 0·629; *P* < 0·001); Fe and Mn (*r* = 0·545; *P* < 0·001); Fe and P (*r* = 0·543; *P* < 0·001); P and Se (*r* = 0·502; *P* < 0·001).

**Table 2.** Mineral concentrations in breast milk by maternal diet pattern (Median and quartiles; mean values and standard deviations)

Mineral (units)			By maternal diet pattern						
	Overall (*n* 63)		Vegan (*n* 23)		Vegetarian (*n* 19)		Omnivore (*n* 21)		
	Median	Quartile 1, quartile 3	Median	Quartile 1, quartile 3	Median	Quartile 1, quartile 3	Median	Quartile 1, quartile 3	*P*
Ca (mg/l)	223	195, 255	229	192, 244	223	206, 271	222	188, 255	0·90
Cu (µg/l)	121	66, 184	107	63, 184	123	62, 166	146	79, 232	0·50
I (µg/l)	80	55, 147	62	40, 132	80	63, 147	93	71, 179	0·10
Fe (µg/l)	159	130, 216	150	131, 186	147	126, 227	184	146, 216	0·42
Pb (µg/l)	1·0	0·1, 2·0	1·0	0·1, 2·0	1·0	0·1, 2·0	1·0	1·0, 3·0	0·22
Mg (mg/l)*									
Mean	32		32		34		31		0·55
sd	9		9		12		7		
Mn (µg/l)	3	2, 4	3	2, 4	3	2, 3	3	2, 4	0·88
P (mg/l)	122	110, 133	125	119, 139	120	110, 138	115	108, 126	0·16
K (mg/l)*									
Mean	416		425		407		416		0·55
sd	53		54		45		60		
Se (µg/l)	19	15, 24	19^a^	18, 25	21^a^	18, 26	17^b^	14, 20	0·007
Na (mg/l)	88	66, 139	91	63, 141	93	69, 174	88	65, 113	0·41
Zn (µg/l)	681	311, 1284	1012	80, 1393	480	80, 835	681	382, 988	0·38

Data represent median (quartile 1, quartile 3) evaluated with a Kruskal–Wallis test unless otherwise noted. Values with a different superscript were significantly different using Dunn’s test for multiple comparisons.

* Data represent means and standard deviations evaluated with ANOVA test.

### Relationship with maternal diet using multiple statistical techniques

**Fig. 1.**
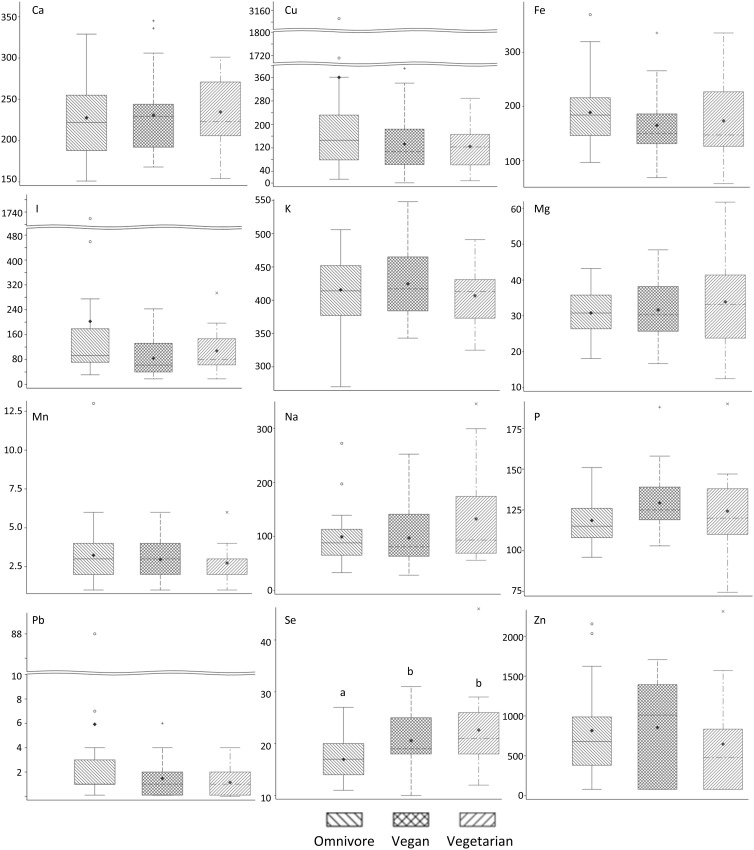
Box and whisker plots showing the distribution of minerals in breast milk by maternal diet pattern. Plots with a different letter are statistically different based on a Kruskal–Wallis analysis (*P* = 0·007) followed by a Dunn’s test for multiple comparisons (omnivore–vegan, *P* = 0·039; omnivore–vegetarian, *P* = 0·008 and vegan–vegetarian, *P* = 0·42).

Table 2 and Fig. 1 provide descriptive statistics and visual plots by maternal diet pattern. The only mineral with significant differences among the diet groups was Se (Kruskal–Wallis, *P* = 0·007). Using Dunn’s method for pair-wise comparisons, median (quartile 1, quartile 3) Se composition was significantly higher (*P* = 0·008 and 0·039, respectively) in milk from vegetarians (21; 18, 26 µg/l) and vegans (19; 18, 25 µg/l) compared with omnivores (17; 14, 20 µg/l). No statistically significant difference in Se between vegetarians and vegans was observed (*P* = 0·42). I was the next mineral approaching significant differences by maternal diet (*P* = 0·10).

Variables used in the machine learning analyses included concentrations for twelve minerals, the use of multi-micronutrient or prenatal supplements (Y/N), maternal age, infant age and maternal BMI. RFI was initially employed considering all sixteen variables and three diet categories, that is, vegan, vegetarian and omnivore. The accuracy, Cohen’s kappa statistic and estimate of error rate for the RFI model were 40·1 %, 0·10 and 63·5 %, respectively. Considering the poor results, a new analysis was performed to include only the most relevant variables identified in the initial RFI model (i.e. se and BMI) and two classification groups: omnivore and plant-based diet. Accuracy, Cohen’s kappa statistic and estimate of error rate significantly improved to 73·2 %, 0·41 and 25·4 %, respectively.

**Fig. 2.**
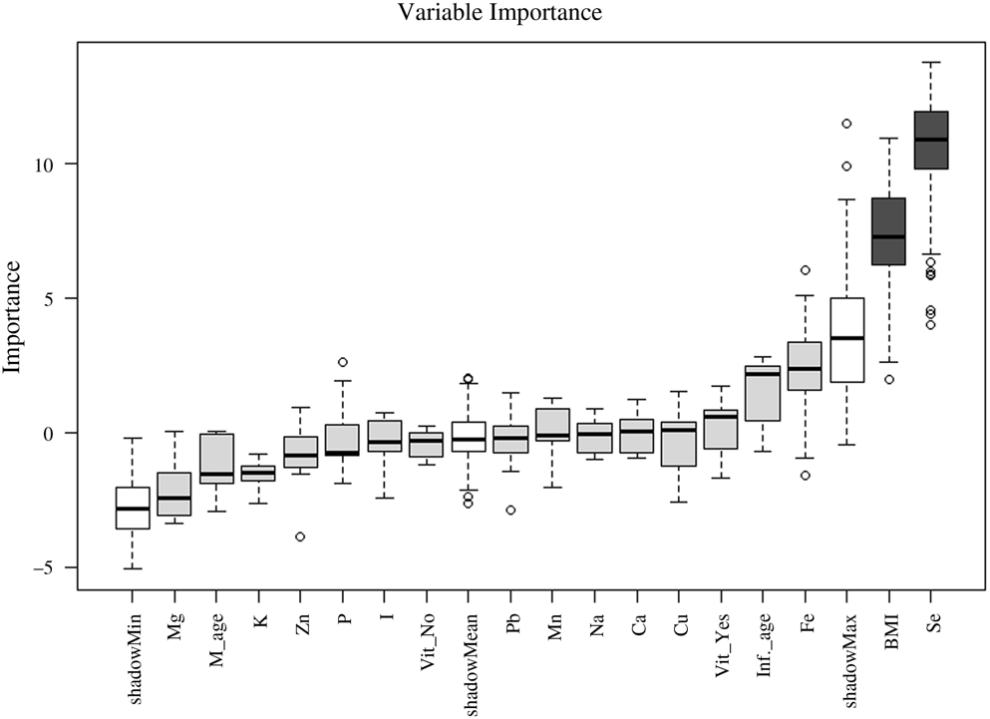
Feature ranking with Boruta (algorithm based on random forests) showing milk Se levels and mother’s BMI are important to identify the different types of diet (omnivore or plant-based). Inf_age, age of infant when sample collected; M_age, maternal age; Vit_No, did not use multi-micronutrient or prenatal vitamin; Vit_Yes, did use a multi-micronutrient or prenatal vitamin.

**Fig. 3.**
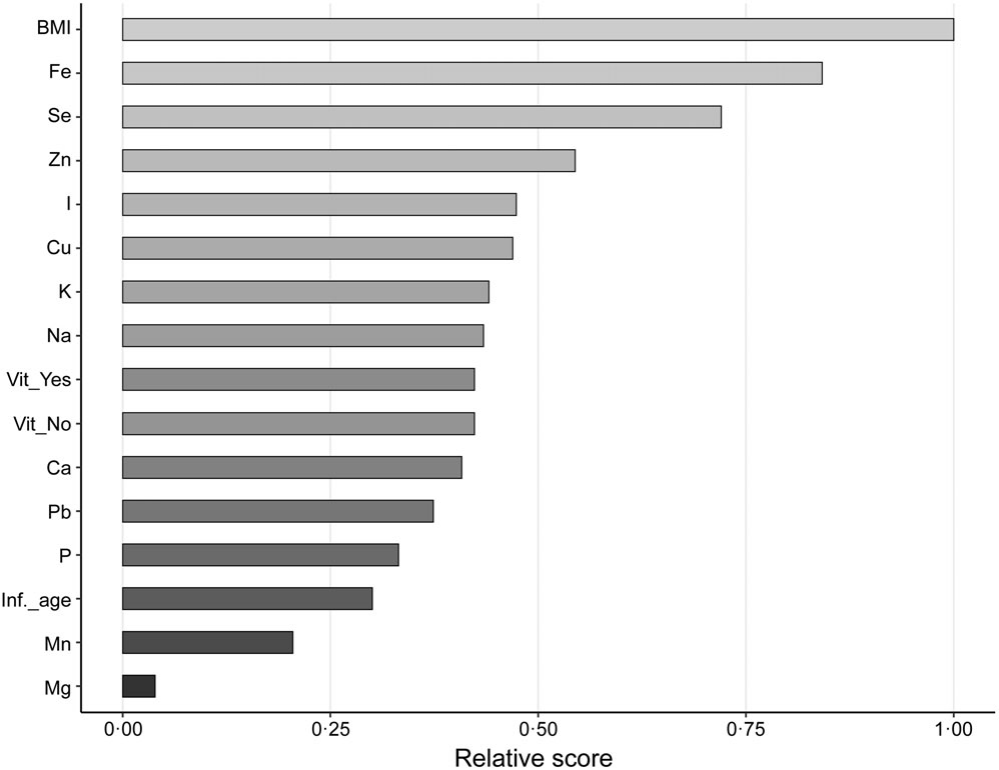
ReliefF algorithm showing BMI and Fe as the most imporant features to identify the different types of diet and their effect on milk elemental concentrations. Se and Zn present moderate importance for classifying the different diets (omnivore *v*. plant-based). Inf_age, age of infant when sample collected; Vit_No, did not use multi-micronutrient or prenatal vitamin; Vit_Yes, did use a multi-micronutrient or prenatal vitamin.

Because no single statistical method is capable of providing a perfect model for real samples, four additional techniques were applied to the data set. As shown in Fig. 2, the Boruta algorithm confirmed the RFI results by selecting se and BMI as the most significant variables for identifying a maternal diet as omnivore or plant-based. The ReliefF method again confirmed the RFI results, although it ranked BMI, Fe and se as the top three most important variables (Fig. 3). The top five variables ranked by the SVM-RFE method in decreasing order were se, Fe, P, I and Cu. Finally, the penalised logistic regression with Lasso penalty identified se, I and Fe as important variables, with coefficient values of −1·28, 1·13 and 0·93, respectively. With a model intercept value of −0·90, note that I and Fe are relatively higher (positive coefficients), and se is relatively lower (negative coefficients) for omnivores compared with individuals following a plant-based diet.

## Discussion

We assessed breast milk mineral composition from US women following plant-based (vegan and vegetarian) and omnivore diets and found that Se was consistently identified as differing using a variety of statistical and machine learning techniques. This approach strengthens our conclusion that Se may be higher in breast milk from women in the USA following a plant-based diet than those following an omnivore diet. Our finding that Se is higher in the milk of women following a plant-based diet agrees with findings by Debski *et al.* in milk collected from lacto-ovo-vegetarians (*n* 26) and omnivores (*n* 12)^(11)^ but is surprising given reports of low Se intake in adults following a vegan diet^(3,29)^. It is possible that the high rate of supplement use in our study population influenced the breast milk Se concentrations we observed, as Se from supplements has been shown to influence breast milk Se concentrations^(30)^. We did not collect detailed information about supplements used or their Se content. To better understand the Se status of lactating individuals following different diet patterns, future studies should also measure maternal serum Se concentrations.

There is limited information regarding composition of other minerals in breast milk based on maternal diet pattern. Finley *et al.* conducted a semi-longitudinal study of 222 samples of milk collected from fifty-two well-nourished participants between 1 and 20 months postpartum. Individuals were classified as vegetarian (*n* 26) if they consumed no meat and only consumed fish no more than twice per month; semi-vegetarian (*n* 6) if they consumed fish more frequently and non-vegetarian (*n* 20) if they consumed meat^(10)^. When comparing milk composition between vegetarians and non-vegetarians, they reported no difference in Ca, Cu, Fe, Mg, K, Na and Zn. Using traditional statistical analysis, we also found no significant difference in these seven minerals by maternal diet pattern, along with no difference in I, Pb, Mn and P. However, using multiple machine learning techniques to address the limitations of a relatively small sample size, Se was repeatedly identified as a predictor of maternal diet-pattern, with Fe and I showing some marginal importance.

Our finding that maternal plant-based diet patterns may influence the Se composition of breast milk warrants further investigation as a potential factor for predicting infant nutritional exposure. Future research should distinguish maternal mineral intake from diet *v*. supplementation, and maternal mineral status, which we were unable to do in this study. A broader study with a larger number of samples may identify other minerals such as I as important breast milk biomarkers for maternal diet. While several of our machine learning models identified Fe as a potential predictor of maternal diet pattern, this is not supported in the literature and highlights the importance of assessing maternal intake and status of micronutrients in addition to milk composition^(30,31)^.

Longitudinal studies suggest that many minerals decline in breast milk for the first 1–2 months postpartum, including Ca, Cu, I, Fe, K, P and Zn^(12,32)^. In our cross-sectional study, the only early postpartum change we observed was a weak negative correlation between Zn and infant age. The youngest infant age in our study was 3·5 weeks postpartum, suggesting we may not have captured the early postpartum window where major time-related changes in mineral composition would be expected. Other minerals, such as Na and Se, have been reported to increase in late lactation stages, likely due to gradual weaning and involution of the mammary gland^(12,32)^. In a longitudinal cohort of breast-feeding women, significant increases in breast milk Na concentration were not observed until 15 months postpartum when daily breast-feeding sessions had dropped below five, suggesting that breast-feeding intensity more than lactation stage influences involution biomarkers^(33)^. We did not measure breast-feeding frequency, which would be valuable to consider in future studies to probe for a potential weaning-effect. It is unclear whether our findings of higher Se in milk from vegetarians compared with omnivores are primarily related to differences in diet or lactation stage between the two groups. This would not likely explain differences in Se concentrations between vegans and omnivores, as lactation stage did not differ in our study between these groups.

Limitations to our study include the cross-sectional design, which did not capture some of the time-dependent changes in breast milk previously reported in the literature. Breast-feeding frequency and breast milk volume were not collected, which might have helped identify compositional changes associated with weaning^(34)^. We did not assess mineral intake from food or supplements. Our sample size (*n* 63) was small and had limited racial diversity; however, it was larger than the two previous studies on minerals in the breast milk from vegan and vegetarian mothers. Additionally, the average breast-feeding duration of our study population was approximately 10 months postpartum, which is higher than US breast-feeding rates (55·8 % of infants receive breast milk at 6 months postpartum)^(35)^ and may limit generalisability.

This study used traditional statistics and machine learning techniques to minimise the sample size limitation (e.g. the RFI model was applied with a 5-fold cross validation and 10 repetitions) and consistently highlighted the importance of Se to differentiate breast milk composition by maternal plant-based diet pattern. It is important to emphasise that no single machine learning model will be perfect at finding all relevant variables, hence the need for applying several methods and using their results to reach better conclusions. In addition to Se, which has been identified as an important variable by all methods evaluated here, Fe was identified as important by three (ReliefF, SVM-RFE and penalised logistic regression with Lasso penalty) out of five machine learning methods, and I by two (SVM-RFE and penalised logistic regression with Lasso penalty) out of five. Therefore, even though no statistical difference has been found for Fe and I using descriptive statistics, the machine learning results suggest these elements should be further investigated. Future research on the impact of maternal plant-based diet on breast milk composition is warranted, using a larger sample size and a longitudinal design. Future studies should also consider maternal mineral intake, maternal mineral status and infant outcome measures including growth and development.

## Supplementary material

For supplementary material accompanying this paper visit https://doi.org/10.1017/S0007114522004007. click here to view supplementary material
