# Supplementary material for: Major and trace mineral composition of milk from lactating women following vegan, vegetarian and omnivore diets
Source: Br J Nutr. 2022 Dec 23;130(6):1005–12. doi: 10.1017/S0007114522004007 (PMC10442794; doi:10.1017/S0007114522004007)
Supplement: Supplementary file 1 [file S0007114522004007sup001.docx]

**Supplementary Material**

**Major and Trace Mineral Composition of Milk from Lactating Women Following Vegan, Vegetarian, and Omnivore Diets**

Maryanne T. Perrin;^1^ Roman Pawlak;^2^ Nicholas Judd;^3^ Jessica Cooper;^1^ George L. Donati^3^

^1^Department of Nutrition, University of North Carolina Greensboro, Greensboro, North Carolina

^2^Department of Nutrition Science, East Carolina University, Greenville, North Carolina

^3^Department of Chemistry, Wake Forest University, Winston-Salem, North Carolina

**Table S1.** Instrumentation and operating conditions used to determine the mineral composition of breast milk.

| **Instrument** | **Instrumental parameter** | **Operating condition** |
| --- | --- | --- |
| ICP-MS/MS | Radio frequency (RF) applied power (W) | 1550 |
|  | Sampling depth (mm) | 10.0 |
|  | Carrier gas flow rate (L/min) | 1.05 |
|  | Collision/reaction cell gas (mL/min) | He (3.5) or H_2_ (4.0) |
|  | Analytes (mass-to-charge ratio, *m/z*) | As (75), Cd (111), Cr (52), Cu (63), Fe (56), I (127), Mn (55), Mo (95), Pb (208), Se (78), Zn (66) |
| ICP-OES | Radio frequency (RF) applied power (W) | 1200 |
|  | Nebulization gas flow rate (L/min) | 0.70 |
|  | Plasma gas flow rate (L/min) | 12.0 |
|  | Auxiliary gas flow rate (L/min) | 1.00 |
|  | Analytes (wavelength, nm) | Ca (396.847), K (766.491), Mg (279.553), Na (589.592), P (213.618) |

**Table S2.** Accuracy evaluation for the determination of trace elements in breast milk by ICP-MS and ICP-OES.

| **Analyte** | **Instrumental method** | **Spike concentration (µg/L)** | **Sample** | **% Recovery** |
| --- | --- | --- | --- | --- |
| As | ICP-MS | 20 | F2 | 102 |
|  |  |  | G11 | 99.5 |
| Ca (II)* | ICP-OES | 4,000 | F2 | 97.3 |
|  |  |  | G11 | 100 |
| Cd | ICP-MS | 20 | F2 | 98.3 |
|  |  |  | G11 | 97.3 |
| Cr | ICP-MS | 40 | F6 | 97.3 |
|  |  |  | G14 | 97.5 |
| Cu | ICP-MS | 40 | F6 | 93.1 |
|  |  |  | G14 | 95.9 |
| Fe | ICP-MS | 40 | F6 | 99.6 |
|  |  |  | G14 | 105 |
| I | ICP-MS | 20 | F2 | 100 |
|  |  |  | G11 | 96.8 |
| K (I)* | ICP-OES | 4,000 | F2 | 95.3 |
|  |  |  | G11 | 98.0 |
| Mg (I)* | ICP-OES | 4,000 | F2 | 100 |
|  |  |  | G11 | 102 |
| Mn | ICP-MS | 20 | F2 | 97.9 |
|  |  |  | G11 | 98.8 |
| Mo | ICP-MS | 20 | F2 | 98.7 |
|  |  |  | G11 | 94.5 |
| Na (I)* | ICP-OES | 4,000 | F2 | 97.5 |
|  |  |  | G11 | 99.4 |
| P (I)* | ICP-OES | 4,000 | F2 | 97.3 |
|  |  |  | G11 | 101 |
| Pb | ICP-MS | 20 | F2 | 96.7 |
|  |  |  | G11 | 96.3 |
| Se | ICP-MS | 20 | F2 | 104 |
|  |  |  | G11 | 106 |
| Zn | ICP-MS | 40 | F6 | 95.7 |
|  |  |  | G14 | 108 |

*I and II represent atomic and ionic emission lines, respectively.

**Table S3.** Limits of detection (LOD) and quantification (LOQ) for ICP-MS and ICP-OES determination of trace elements in milk calculated according to IUPAC recommendations as three (or ten) times the standard deviation of the blank (*S_bl_*, n = 15) divided by the calibration curve slope (*m*), i.e. LOD = 3*S_bl_* / *m*, and LOQ = 10*S_bl_* / *m*. Values are calculated for concentrations in the original sample, before any dilution.

| **Analyte** | **Instrumental method** | **LOD (µg/L)** | **LOQ (µg/L)** |
| --- | --- | --- | --- |
| As | ICP-MS | 0.3 | 1 |
| Ca (II)* | ICP-OES | 70 | 200 |
| Cd | ICP-MS | 0.2 | 0.7 |
| Cr | ICP-MS | 0.9 | 3 |
| Cu | ICP-MS | 0.6 | 2 |
| Fe | ICP-MS | 2 | 10 |
| I | ICP-MS | 1 | 4 |
| K (I)* | ICP-OES | 30,000 | 100,000 |
| Mg (I)* | ICP-OES | 1,000 | 4,000 |
| Mn | ICP-MS | 0.2 | 0.7 |
| Mo | ICP-MS | 0.3 | 1 |
| Na (I)* | ICP-OES | 7,000 | 23,000 |
| P (I)* | ICP-OES | 4,000 | 15,000 |
| Pb | ICP-MS | 0.1 | 0.4 |
| Se | ICP-MS | 0.4 | 1 |
| Zn | ICP-MS | 80 | 300 |

*I and II represent atomic and ionic emission lines, respectively.
